# Emergence of transmissible *mcr-9.1* plasmids in clinical *Cronobacter sakazakii*: CRISPR typing unravels phage-driven evolution and high-risk lineage

**DOI:** 10.1128/aem.01379-25

**Published:** 2025-09-02

**Authors:** Haiyan Zeng, Huanhuan Zou, Xiaoyi Huang, Bingshao Liang, Shenshen Liu, Wenxin Zhao, Min Xu, Moutong Chen

**Affiliations:** 1 School of Biomedicine and Pharmaceutical Science, Guangdong University of Technology 47870, Guangzhou, China; 2 Guangzhou Women and Children’s Medical Center, Guangzhou Medical University, Guangdong Provincial Clinical Research Center for Child Health 159390, , Guangzhou, China; 3 Department of Laboratory Medicine, the First Affiliated Hospital, Zhejiang University School of Medicine 26441, , Hangzhou, China; 4 Guangdong Provincial Key Laboratory of Microbial Safety and Health, State Key Laboratory of Applied Microbiology Southern China, Institute of Microbiology, Guangdong Academy of Sciences 514144, Guangzhou, China; Universita degli Studi di Napoli Federico II, Portici, Italy

**Keywords:** *Cronobacter*, CRISPR, *mcr-9.1*, ST13, ST256, IncHI2, IncFIB

## Abstract

*Cronobacter* spp. is an important foodborne pathogen that causes serious neonatal infections. The prevalence of the colistin resistance gene *mcr-9* in *Cronobacter* spp. strains isolated from foods has been reported; however, there are no reports for isolates from clinical infections. Surveillance of *Cronobacter* infection was conducted at a prominent children’s hospital in China during 2015–2023 and also in a tertiary hospital in 2023. There were a total of seven *Cronobacter sakazakii* infection cases, and *mcr-9.1* was detected in one ST13 and two ST256 strains. After adding the publicly available sequence data, the backbones of IS*1R-mcr-9.1-wbuc-qsec* in *C. sakazakii* ST13 and IS*903B-mcr-9.1-wbuc*-IS*26* in ST256 strains are proposed to be associated with IncFIB and IncHI2 plasmids, respectively. More importantly, the *mcr-9.1*-positive IncHI2 plasmid in *C. sakazakii* ST256 clinical strain can be transferred to *Escherichia coli* J53, conferring multiple drug resistance. These ST13 and ST256 strains were assigned to 19 CRISPR types (CTs), and *mcr-9.1*-positive strains belonged to specific CTs in China. While ST256 has not been detected in contaminated food, its presence in insect and environmental samples suggests a potential transmission route requiring further investigation. Combined phylodynamic and CRISPR spacer analyses suggested phage interactions shaped *C. sakazakii* ST13 evolution. The relatively high-risk lineage bridging clinical (*mcr-9.1*-positive CT212) and food chain (CT208) reservoirs displayed CRISPR adaptation signatures: selective type 1 ancestral spacer loss coupled with type 2 ancestral preservation. Plasmid-mediated *mcr-9.1* gene transfer in *C. sakazakii* poses a potential threat to public health, thereby necessitating vigilant clinical monitoring.

**IMPORTANCE:**

*Cronobacter* spp. is ubiquitous in food and the environment, although the infection incidence of this pathogen is low, the case-fatality rate is high in premature and immunocompromised infants. In this study, we first report that the silent dissemination of *mcr-9.1* in *Cronobacter sakazakii* ST13 and ST256 strains was associated with IncFIB and IncHI2 plasmids, respectively. The *mcr-9.1*-encoding IncHI2 plasmid was transferable from the ST256 strain, suggesting significant dissemination potential among clinically relevant bacteria. Taking into account the temporal organization of spacers, we first used CRISPR diversity to trace the origins of *mcr-9.1*-positive *C. sakazakii* strains and deduce their divergent evolution through the preserved spacer information targeting specific phages. Our CRISPR-enhanced molecular epidemiology approach enables robust phylogenetic inferences despite limited clinical isolates, demonstrating a transferable framework applicable to other emerging pathogens with surveillance data.

## INTRODUCTION

*Cronobacter* spp. is an important foodborne pathogen associated with the cases of life-threatening necrotizing enterocolitis, meningitis, and sepsis in neonates and infants (1, 2). This genus is ubiquitous in food and the environment, and *Cronobacter sakazakii* is the most important pathogenic species (3–5). Although the incidence of *Cronobacter* spp. infection is low, the fatality rate is really high in premature and immunocompromised infants (1, 2, 6). In comparison to other members of the *Enterobacteriaceae* family, *Cronobacter* spp. is more susceptible to the majority of commonly utilized antibiotics (7–9).

Colistin serves as one of the final antibiotic options for treating infections caused by carbapenem-resistant *Enterobacterales*, and various bacterial species have been found to possess the colistin resistance genes, ranging from *mcr-1* to *mcr-10* (10–15). *mcr-9* is one of the most prevalent subtypes of the *mcr* family worldwide (11, 13, 16). Our previous study also reported a silent prevalence of *mcr-9* in various bacterial genera, circulating in the form of three distinct backbone types (12). Compared to those strains carrying *mcr-1*, strains harboring *mcr-9* exhibit great sensitivity to colistin due to its weak activity and low expression level (17, 18). However, Song et al. reported *mcr-9* exhibited a unique propensity for transmission within the *Enterobacter*, and *Enterobacter* strains exhibiting high expression of *mcr-9* and resistance to colistin had emerged, signaling a considerable threat of *mcr-9* evolving into a widespread and pervasive “true resistance gene” (18).

The sporadic detections of *mcr* genes in *Cronobacter* spp. have been reported in previous studies (9, 19–21). Recently, Zhu et al. found that 133 of 877 previously undescribed *Cronobacter* strains were carrying *mcr* genes in a large-scale genomic survey of food isolates. Notably, 130 of these strains carried the *mcr-9.1* gene (22). Moreover, they discovered two core flanking structures of *mcr-9.1* among many STs (22). Nevertheless, while multiple studies have investigated the occurrence of *mcr-9.1* in *Cronobacter* foodborne isolates, clinical reports remain absent due to the pathogen’s low infection incidence. The potential epidemiological links between clinical and foodborne *mcr-9-*positive strains remain unexplored. Notably, CRISPR typing demonstrates superior discriminatory power over 7-loci MLST in differentiating closely related *Cronobacter* strains (23). The observed CRISPR diversity within this genus may serve as a valuable molecular tool for tracing the evolutionary trajectory and transmission pathways of *mcr-9*-positive strains, particularly their emergence and dissemination across clinical and environmental reservoirs.

In this study, we investigated the prevalence and flanking structures of the *mcr-9* gene in clinical *Cronobacter* isolates collected from a long-term surveillance program conducted in two Chinese hospitals. We further experimentally validated the conjugation transfer potential of *mcr-9*-positive plasmid within this genus. Through integration of publicly available genomic data, we reconstructed the phylogenetic origin and evolutionary relationship of *mcr*-9-positive isolates using CRISPR diversity and whole-genome single nucleotide polymorphism (wgSNP) anaylses.

## MATERIALS AND METHODS

### Strain collection, antimicrobial susceptibility profile, and *mcr-9* detection

The surveillance of *Cronobacter* spp. infection was conducted during 2015–2023 at Guangzhou Women and Children’s Medical Center, a prominent hospital located in China as well as in the First Affiliated Hospital, Zhejiang University School of Medicine during 2023. The suspected bacterial strains were identified by using an automated VITEK 2 Compact system (bioMérieux, Marcy l’Etoile, France). The antimicrobial susceptibility profiles of *Cronobacter* spp. isolates were determined by disk diffusion method or broth microdilution method (1, 6). Genomic DNA was extracted with the HiPure Bacterial DNA Kit (Magen Technologies, Guangzhou, China). The STs were identified through the PubMLST database (https://pubmlst.org/organisms/cronobacter-spp). The PCR detection of *mcr* genes was performed according to a previous study (11).

### Whole genome sequencing and analyzation of the flanking structure of *mcr-9.1* genes

All samples isolated from Guangzhou Women and Children’s Medical Center were subjected to 2 × 250 bp paired-end sequencing by the Hiseq 2500 instrument (Illumina), while three isolates were also sequenced by the PacBio RS II (Pacific Biosciences). The assembly and annotation were performed as described in a previous study (24). All strains with whole-genome sequences belonging to the same STs as the *mcr*-positive strain in this study were downloaded from the *Cronobacter* PubMLST database (https://pubmlst.org/organisms/cronobacter-spp), NCBI GenBank database, and the National Genomics Data Center, Beijing Institute of Genomics, Chinese Academy of Sciences/China National Center for Bioinformation (https://ngdc.cncb.ac.cn/gwh) (22). These sequences were chosen to detect the prevalence of *mcr* genes. The plasmid sequence or contig/scaffolds harboring *mcr* genes were used for the flanking structure analyses. The types of plasmids were determined by PlasmidFinder 2.1 (25) and blastn result in NCBI Non-Redundant Database. Linear comparisons of *mcr* genes and flanking genetic context among the same ST strains were visualized by Easyfig 2.2.3 (26).

### Conjugation analysis and minimum inhibitory concentrations (MIC) determination

The ability of *C. sakazakii* GZcsf-1 to transfer plasmid pGW1 with beta-lactam, tetracycline, and *mcr-9* resistance genes to other bacteria was investigated by conjugation experiments using *E. coli* J53 as recipients according to a previous study with slight modification (27). Briefly, donor and recipient strains were mixed at ratios of 1:1, 7:3, and 4:1 and then selected on LB Agar plates supplemented with tetracycline (8 µg/ml) and rifampicin (128 µg/ml), respectively. PCR assays targeting *mcr-9* were performed for all potential transconjugants using primers as described in a previous study (11). The antimicrobial susceptibility of *C. sakazakii* GZcsf-1, *E. coli* J53, and transconjugant *E. coli* pGW1-J53 was tested by the broth dilution method and interpreted as per CLSI guidelines.

### CRISPR typing and visualization of CRISPR arrays

The identification of CRISPR arrays, extraction of spacers, and determination of CRISPR types (CTs) were performed according to our previous study (23). The protospacers of the CRISPR array spacer sequences were searched by blastn. The visualization and comparison of CRISPR array spacers from the same ST strains were implemented using CCTK CRISPRdiff (28).

### Phylogenetic tree construction and temporal analysis

The wgSNP among one ST strain was calculated by Harvest (29) and extracted by SNP-sites software (30), then the maximum likelihood (ML) phylogenetic tree based on wgSNP sites was constructed using FastTree software (31). All the phylogenetic trees were edited in iTOL (32). A root-to-tip regression analysis was performed by TempEst 1.5.3 to assess the reliability of the temporal signal in the ML tree (33). After deleting the questionable sequences, a Bayesian phylogenetic approach was used to estimate the nucleotide substitution rates and divergence times of *C. sakazakii* lineages by BEAST v1.10 according to our previous studies (6, 24, 34).

## RESULTS

### *Cronobacter* infection cases and *mcr-9* gene detection

Five *Cronobacter*-infected infant cases and two *Cronobacter*-infected adult cases were monitored in two hospitals of China. Five *C. sakazakii* strains were isolated from infant samples (brain abscess fluid, blood, ascites, and midstream urine), one *Cronobacter malonaticus* from an adult bile sample, and one *C. sakazakii* strain from adult ascites, as detailed in Table 1. *C. sakazakii* GZcsf-1 causing neonatal meningitis and GZfs causing necrotizing enterocolitis had been reported in our previous studies (1, 6). *C. sakazakii* CRZK exhibited an identical multiple antibiotic resistance profile to *C. sakazakii* GZcsf-1, *C. sakazakii* BD was resistant to five antibiotics, *C. malonaticus* H5 and *C. sakazakii* H6 were resistant to cefazolin and amoxicillin/clavulanic, and *C. sakazakii* bq was susceptible to all commonly used antibiotics (Table S1). Although all *Cronobacter* strains exhibited phenotypic susceptibility to colistin, the *mcr-9.1* gene was identified in two *C. sakazakii* ST256 strains and one ST13 strain (Table 1). Comprehensive screening revealed no other *mcr* gene variants (*mcr-1* to *mcr-10*) in these clinical isolates.

**TABLE 1:** Clinical *Cronobacter* spp. strains collected in this study

Strain	Region	Year	Source	Age	Feeding regime	ST	*mcr-9.1^c^*
*C. sakazakii* GZcsf-1^*a*^	Guangzhou	2015	Brain abscess fluid	26 days	Breast	256	+
*C. sakazakii* CRZK^*b*^	Guangzhou	2015	Blood	17 days	Formula	256	+
*C. sakazakii* BD	Guangzhou	2016	Blood	8 days	Unsure	21	−
*C. sakazakii* GZfs^*a*^	Guangzhou	2019	Ascites	17 days	Unsure	64	−
*C. sakazakii* bq	Guangzhou	2022	Midstream urine	41 days	Unsure	13	+
*C. malonaticus* H5	Hangzhou	2023	Ascites	58 years	–	7	−
*C. sakazakii* H6	Hangzhou	2023	Bile	78 years	–	4	−

^*a*^ Strains GZcsf and GZfs (PubMLST IDs: 2635 and 4182) were previously reported (1, 6).

^*b*^ Strain CRZK was isolated from a preterm infant.

^*c*^ The − and + symbols indicated the absence and presence of *mcr-9.1*, respectively.

### Incidence rate and flanking structures of *mcr-9.1* among *C. sakazakii* ST13 and ST256 strains

All available *C. sakazakii* ST13 and ST256 strains in public databases were downloaded to detect the prevalence of *mcr* genes (Table S2). The *mcr-9.1* gene was detected in 11.9% (7/59) of ST13 and 40% (2/5) of ST256 strains, all isolated from China. No other *mcr* genes were detected. Except for three *mcr-9.1*-positive clinical strains identified in this study, all other *mcr-9.1*-positive strains were isolated from food, such as grain food products, powdered infant formula, and beverages (Table 1).

**Fig 1:**
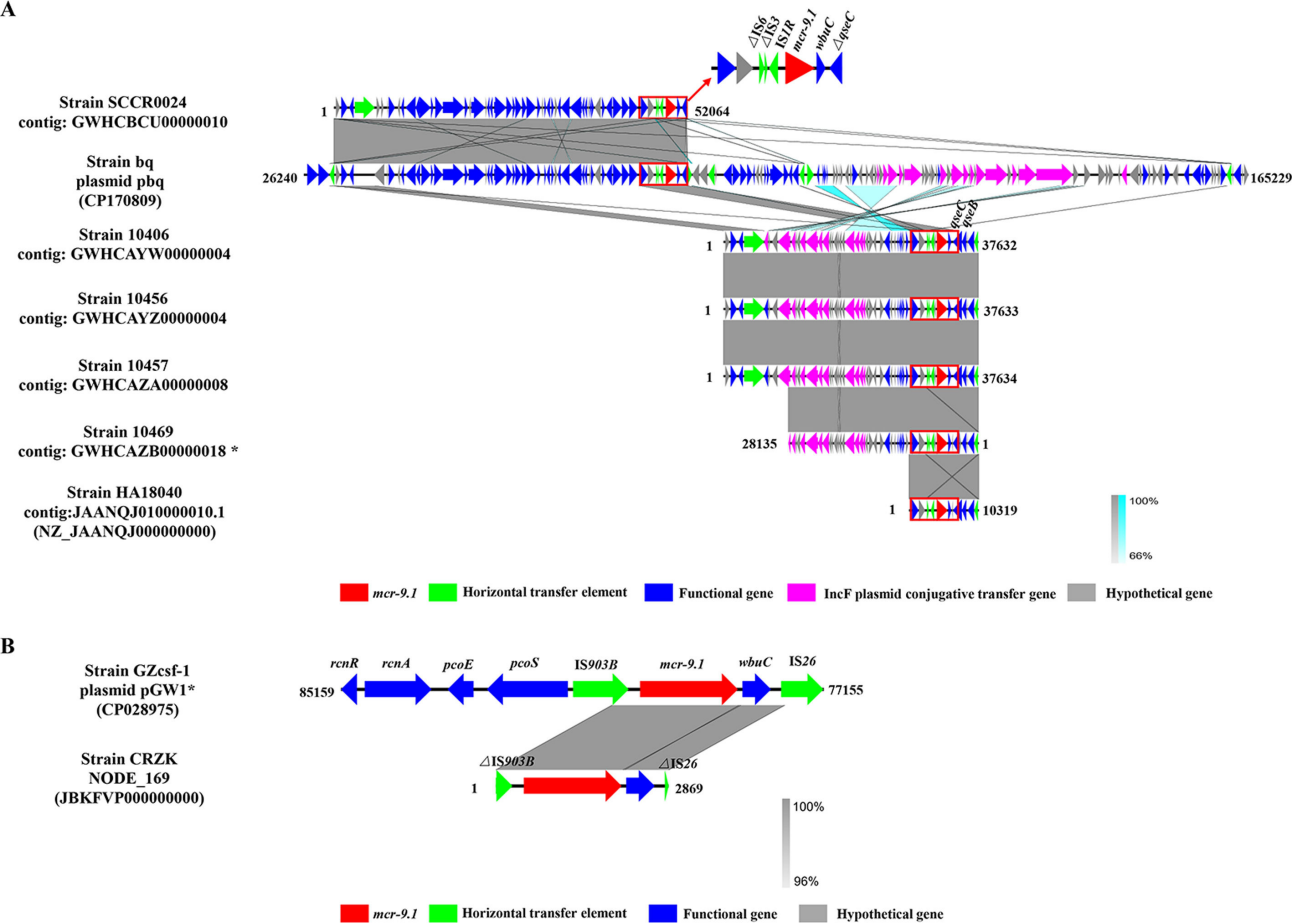
The flanking structures of *mcr-9.1* genes in *C. sakazakii* ST13 (**A**) and ST256 (**B**) strains. △ represents truncated genes.

The flanking structure of uncharacterized protein-hypothetical protein-△*IS6*-△*IS3-IS1R-mcr-9.1-wubC*-△*qseC* (abbreviated as *IS1R-mcr-9.1-wubC-qseC*) was observed in all *C. sakazakii* ST13 strains (Fig. 1A). Indeed, except for strain bq and SCCR0024, the other five strains had intact *qseC*/*qseB* two-component system genes. Plasmid pbq belonged to the IncFIB plasmid based on the blastn result. Many IncF plasmid conjugative transfer proteins were also found in the contigs of strains 10406, 10456, 10457, and 10469, suggesting that these sequences may belong to the IncF plasmid (Fig. 1A). At the same time, the sequences of IncFIB and IncFII plasmid were also detected in 10406, 10456, 10457, 10469, and HA18040 strain by PlasmidFinder 2.1. Combined with these results, this type of *mcr-9.1* backbone is probably associated with IncFIB plasmid.

As shown in Fig. 1B, the genetic structure of *rcnR-rcnA-pcoE-pcoS-IS903B-mcr-9.1-wubC-IS26* in the plasmid pGW1 of *C. sakazakii* GZcsf-1 belonged to the prevalent type 3 of *mcr-9.1* backbone in our previous study (12). △*IS903B-mcr-9.1-wubC*-△*IS26* was also observed in the short contig of *C. sakazakii* CRZK. This strain had the same antibiotic resistance profile as strain GZcsf-1, and all the drug resistance genes in plasmid pGW1 of strain GZcsf-1 were also identified in strain CRZK. Plasmid pGW1 was an IncHI2 type drug resistance plasmid (1). Moreover, the sequences of IncHI2 and IncHI2A plasmid were also detected in strain CRZK by PlasmidFinder 2.1. The core flanking structure of *IS903B-mcr-9.1-wubC-IS26* may be related to IncHI2 plasmid in *C. sakazakii* strain.

### Conjugation analysis of *mcr-9.1*-positive IncHI2 plasmid in *C. sakazakii* ST256 strain

Except *mcr-9.1*, there were another 19 drug resistance genes in IncHI2 plasmid pGW1 of *C. sakazakii* ST256 strain GZcsf-1 (1). In this study, plasmid pGW1 was successfully transferred into *E. coli* J53 via conjugative transfer at all tested donor-to-recipient ratios (1:1, 7:3, and 4:1), yielding the transconjugant strain pGW1-J53 with stable antibiotic resistance profiles (Table 2). As expected, the transconjugant pGW1-J53 was resistant to tetracycline, ampicillin, amoxicillin/clavulanic, ceftriaxone, cefazolin, cefuroxime, trimethoprim/sulfamethoxazole, and gentamicin (Table 2). This multidrug resistance phenotype is mediated by resistance genes other than *mcr-9.1*. The presence of *mcr-9.1* in the IncHI2 conjugative plasmid of *C. sakazakii* underscores a significant risk for rapid future dissemination. The *C. sakazakii* ST13 strain bq, which carried solo drug resistance gene *mcr-9.1* in its IncFIB plasmid pbq and demonstrated susceptibility to all tested antibiotics, was excluded from conjugation experiments owing to the unavailability of selectable antimicrobial resistance markers.

**TABLE 2:** Antimicrobial drug susceptibility profiles of *C. sakazakii* Gzcsf-1, *E. coli* J53, and transconjugant pGW1-J53

Antimicrobial group	Antibiotic	Antimicrobial susceptibility^*a*^		
		GZcsf-1	pGW1-J53	J53
Penicillins	Ampicillin	≥256/R	256/R	2/S
	Amoxicillin/clavulanic	32/R	32/R	4/S
	Piperacillin/tazobactam	≤4/S	≤4/S	≤4/S
Cephalosporins	Cefepime	≤1/S	≤0.12/S	≤0.12/S
	Ceftriaxone	≥64/R	32/R	≤0.25/S
	Cefazolin	≥64/R	≥64/R	≤4/S
	Ceftazidime	2/S	2/S	≤0.12/S
	Cefuroxime	≥64/R	≥64/R	4/S
Aminoglycosides	Gentamicin	256/R	256/R	0.5/S
Carbapenems	Imipenem	≤1/S	≤0.25/S	≤0.25/S
Sulfonamides	Trimethoprim/sulfamethoxazole	≥320/R	≥320/R	≤20/S
Tetracyclines	Tigecycline	≤0.5/S	≤0.5/S	≤0.5/S
	Tetracycline	1024/R	1024/R	2/S
Lipopeptide	Colistin	0.5/S	0.5/S	0.5/S

^*a*^ All results are interpreted using the *Enterobacteriaceae* data from the CLSI of MIC interpretive criteria.

### CRISPR typing and phylogenetic tree analyses of *C. sakazakii* ST13 and ST256 strains

Apart from three ST13 and one ST256 strains that lacked intact CRISPR arrays, the remaining 60 strains were assigned to 19 CTs including 17 new types (Tables S2 and S3). Moreover, *mcr-9.1*-positive strains were also linked to specific CTs, such as CT212, CT219, CT221, and CT222. CT218 was detected in both the *mcr-9.1*-positive strain HA18040 and other *mcr-9.1*-negative foodborne strains.

**Fig 2:**
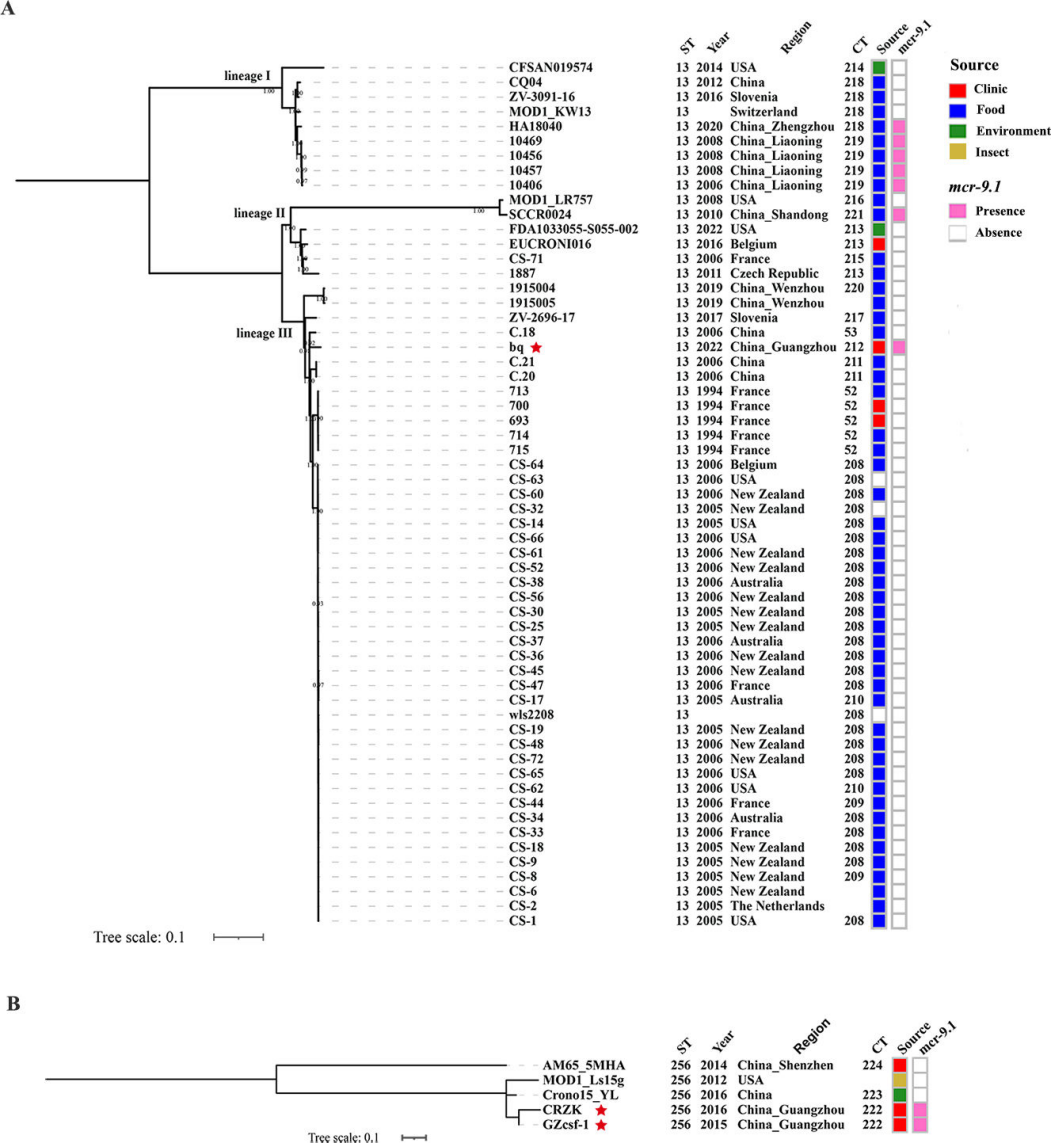
Maximum likelihood phylogenetic tree based on wgSNP of *C. sakazakii* ST13 (**A**) and ST256 (**B**) strains. The information of ST, CT, source, year, region, *mcr-9.1* genes, and two types of ancestral spacers in ST13 CRISPR1 array are listed next to the corresponding strains. The clinical *C. sakazakii* strains isolated in this study are marked with red stars.

There were three lineages I, II, and III observed in this phylogenetic tree (Fig. 2A). The *mcr-9.1*-positive CT218 strain HA18040 and CT219 strains 10406, 10456, 10457, and 10469 were gathered into the distinct lineage I. *mcr-9.1*-positive CT221 strain SCCR0024 maintained the closest relationship with the CT216 strain MOD1_LR757 in lineage II. The *mcr-9.1*-positive CT212 clinical strain bq in this study demonstrated a closest phylogenetic proximity to China-sourced CT53 foodborne strain C.18 (isolated from milk powder), clustered with global strains, constituting the unique lineage III. Importantly, though *mcr-9.1* dissemination spanned all lineages, lineage III (containing the clinical index strain) demonstrated significant expansion dominance.

As for ST256 strains, two *mcr-9.1*-positive CT222 strains GZcsf-1 and CRZK had the closest relationship with CT223 environmental strain Crono15_YL, which has not been reported in any contaminated food, rendering the infection route a mystery (Fig. 2B).

### Diversity of CRISPR spacers in *C. sakazakii* ST13 and ST256 strains

**Fig 3:**
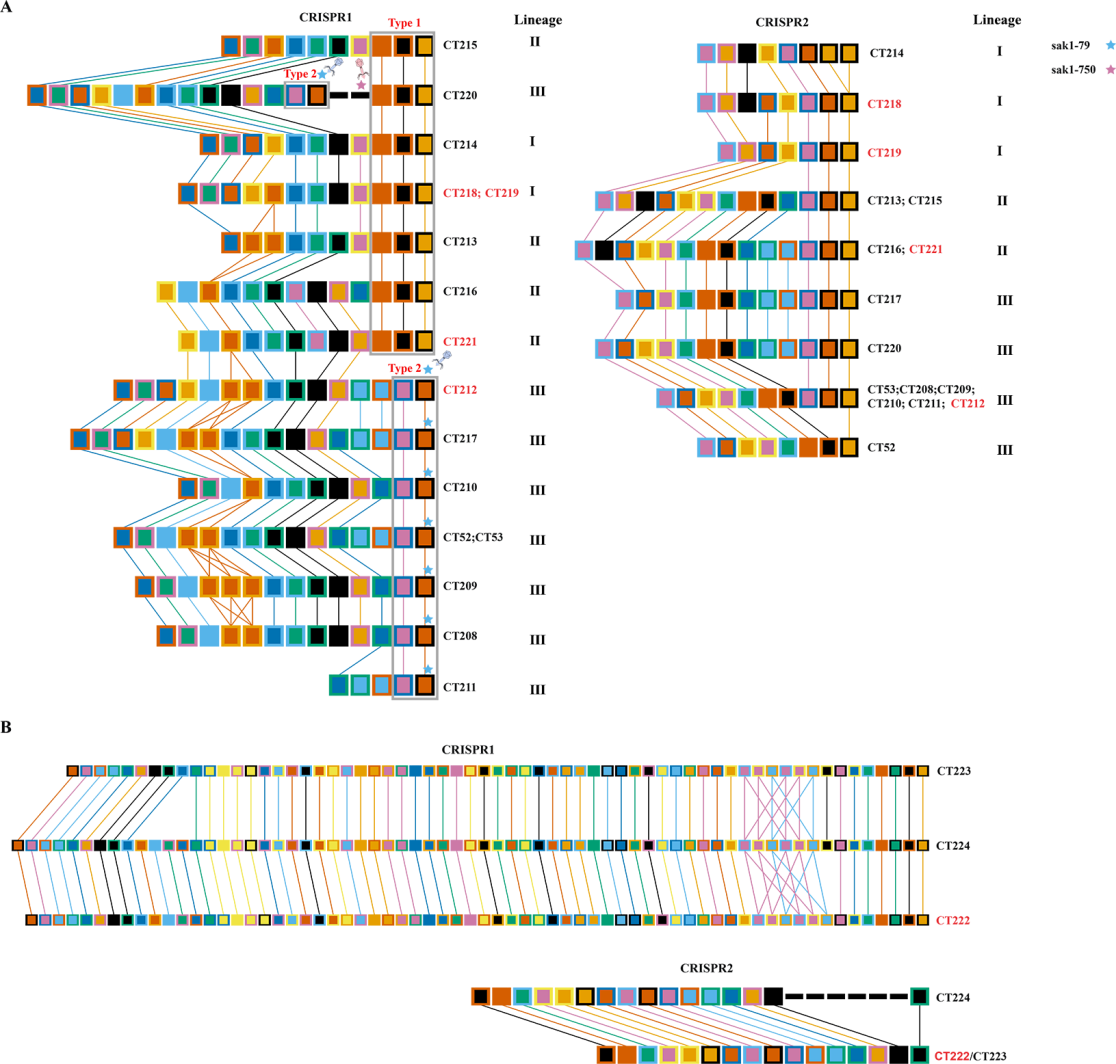
Illustration of the relationship between two CRISPR arrays of *C. sakazakii* ST13 (**A**) and ST256 (**B**) strains. The leader proximal end of each array is on the left and the distal end is on the right. The black bar represents that this spacer is found only in one array. Two types of ancestral spacers in ST13 CRISPR1 arrays are marked with gray boxes. The spacers sak1-79 and sak1-750 targeting the sequences of phages are marked with blue and pink stars, respectively. CT types harboring *mcr-9.1*-positive strains are highlighted in red.

As the polarity of spacers exists in the CRISPR array, new spacers are added to the proximal end, and spacers at the leader distal end are more ancestral and often shared among bacterial common ancestors (23). Among ST13 strains, there were two types of ancestral spacers in the CRISPR1 array (Fig. 3A). Type 1 ancestral spacers were observed among *mcr-9.1*-positive foodborne strains and other five CT strains, type 2 was detected in *mcr-9.1*-positive clinical strain and other seven CTs. Incorporated the type of ancestral spacers in the CRISPR1 array with lineages observed in the phylogenetic tree, lineage I and II had type 1 ancestral spacers, lineage III had type 2 ancestral spacers. The most interesting phenomenon was discovered in CT220 (lineage III). It had two types of ancestral spacers, and the type 1 was more distal to leader, indicating it may be more ancestral than type 2. Regarding CRISPR2 arrays, the oldest ancestral spacer and the newest spacer were identical across all ST13 strains; variation occurred only in the intervening spacers. In contrast to CRISPR1, variation within the CRISPR2 array was less pronounced. Furthermore, lineage II exhibited greater similarity in its CRISPR2 array to lineage III than to lineage I. After examining the targeting sequences (protospacer) of these spacers, spacer sak1-79 in type 2 ancestral spacers targeted the terminase large subunit of *Enterobacter* phage BUCT554, sak1-750 unique in CT220 between two types of ancestral spacers targeted hypothetical protein of *Enterobacter* phage vB_EclS_CobraSix (Fig. 3A and Table S4). The infection of these phages may contribute to the divergent evolution of *C. sakazakii* ST13 strains.

Regarding the ST256 strains, the *mcr-9.1*-positive CT222 strains shared identical CRISPR2 arrays with CT223. Notably, comparative analysis revealed that CT224 harbored six unique spacers absent in both CT222 and CT223. When referenced against CT224, CT222 displayed a loss of one spacer in its CRISPR2 array, whereas CT223 exhibited a more pronounced deletion of four spacers in its CRISPR1 array (Fig. 3B). Despite the presence of the CRISPR3 array in ST256 strains, all strains shared three identical spacers within this array, prompting us to omit its depiction from Fig. 3 (detailed information is shown in Table S3). As shown in Table S4, two spacers from CRISPR1 targeted distinct regions within the *E. coli* strain 7/2 plasmid p7_2.2, while seven spacers targeted different regions of the *C. sakazakii* strain MOD1-GK1025B plasmid pGK1025B_3. In addition, one spacer from CRISPR2 targeted the *Enterobacter roggenkampii* strain WCHER090065 plasmid pMCR10_090065. The presence of these spacers in all ST256 strains suggests that infection by these similar plasmids was part of the evolution history.

### Evolutionary history of *C. sakazakii* ST13 strains inferred by phylodynamic analyses and ancestral spacers information

**Fig 4:**
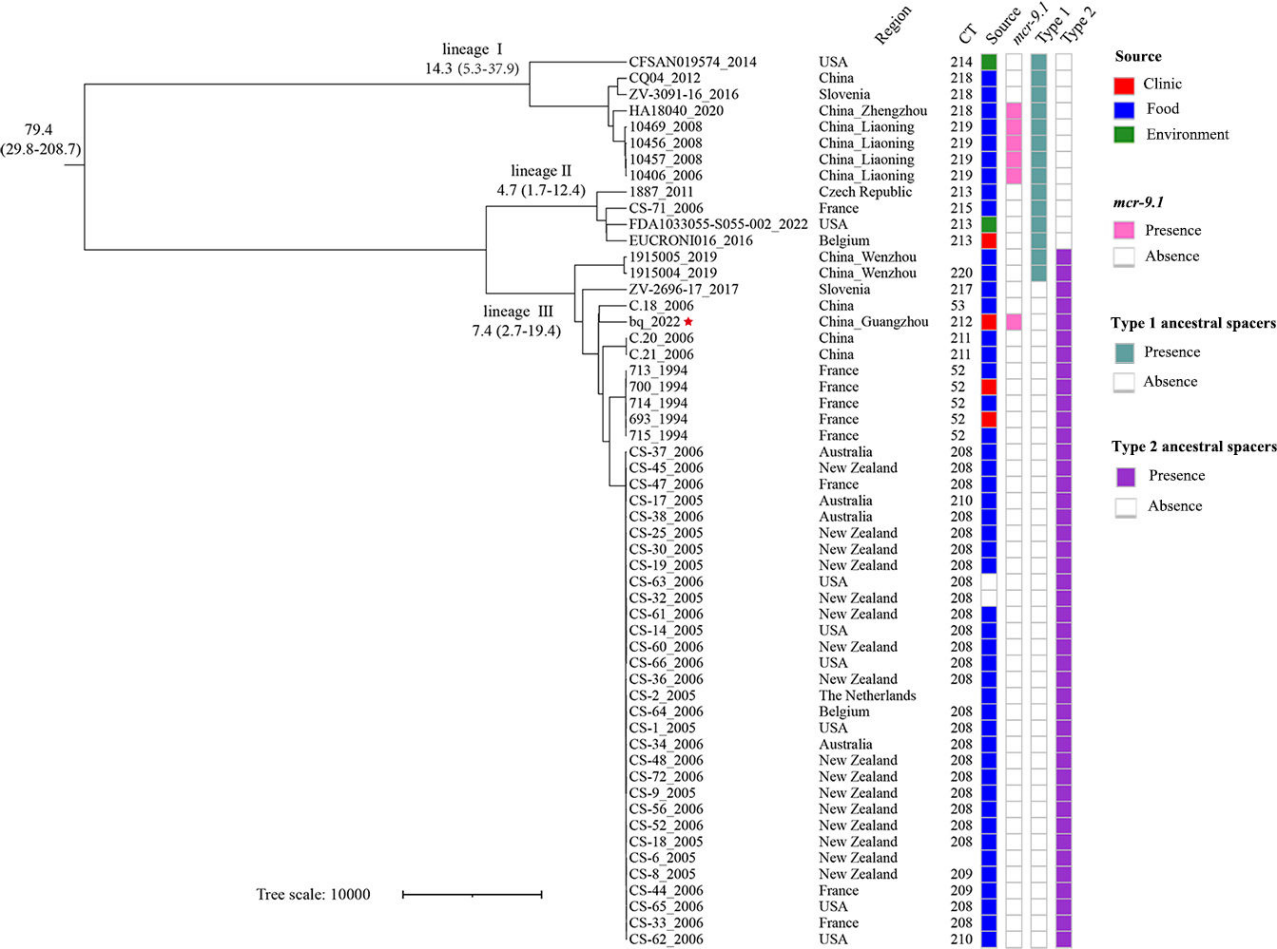
Timed phylogeny of *C. sakazakii* ST13 strains in the maximum clade credibility tree, measured in thousands of years before the present. The information of ST, CT, source, year, region, *mcr-9.1* genes, and two types of ancestral spacers in ST13 CRISPR1 array are listed next to the corresponding strains. The clinical *C. sakazakii* strains isolated in this study are marked with red stars.

Two strains, strain MOD1_LR757 and *mcr-9.1*-positive strain SCCR0024 in lineage II, were not chosen for temporary analysis as they were identified as distant outliers in our phylogenetic assessment using TempEst 1.5.3 (33). This software evaluates temporal signal reliability in sequence data—a critical step before molecular clock analysis. The phylodynamic analysis of *C. sakazakii* ST13 was then performed by BEAST software (Fig. 4). The nucleotide substitution rate of *C. sakazakii* ST13 strains was 5.39 × 10^−6^ substitutions/site/year (95% confidence interval [CI], 1.0 × 10^−6^–9.3 × 10^−6^), which had placed the most recent common ancestor (tMRCA) of this ST strains 79.4 (95% CI, 29.8–208.7) thousand years ago. The lineage I, II, and III was originated from 14.3 (95% CI, 5.3–37.9), 4.7 (95% CI, 1.7–12.4), and 7.4 (95% CI, 2.7–19.4) thousand years ago, respectively. When incorporated into phylodynamic tree, the ancestral spacers types in the CRISPR1 array revealed a key distinction: all strains in lineage I and II possessed type 1 ancestral spacers, whereas all strains in lineage III possessed type 2 ancestral spacers. Notably, strain 1915004 (CT220) harbored both type 1 and type 2 ancestral spacers. Its closest phylogenetic relative, strain 1915005, exhibited a truncated CRISPR1 array due to limited sequencing coverage, retaining partial type 1 ancestral spacers alongside intact type 2 ancestral spacers. The phylogenetic reconstruction clustered both strains within distinct subclades at the phylogenetic periphery of lineage III, suggesting early divergence within this lineage. These findings suggest that lineage III, including *mcr-9.1*-positive clinical strain, likely descended from an ancestral progenitor carrying two ancestral spacer types, with subsequent selective shedding of type 1 ancestral spacers conferring significant evolutionary fitness benefits that facilitated its clonal expansion.

## DISCUSSION

*Cronobacter* is an important foodborne pathogen that is ubiquitous in food and the environment (3, 4, 7, 9). Despite the relatively low incidence of *Cronobacter* infection, the fatality rates among premature and immunocompromised infants range from 40 to 80% (35). During the surveillance period in this study, there were a total of five infant cases of *C. sakazakii* infection, with unfortunately two infants succumbing to the infection (1, 6). Another two adult patients in this study exhibited mild symptoms of infection, consistent with a non-severe inflammatory condition. Meanwhile, *mcr-9.1* was detected in three *C. sakazakii* isolates, including ST13 strain bq and ST256 strains GZcsf-1 and CRZK. After comprehensive integration of publicly available strain data, 11.9% (7/59) of ST13 strains and 40% (2/5) of ST256 strains were detected to carry *mcr-9.1*.

In accordance with the previous paper, there were two types of core flanking structures of *mcr-9.1* among *C. sakazakii* strains (22). Furthermore, we first infer that the core flanking structure of IS*1R-mcr-9.1-wbuc-qsec* in *C. sakazakii* ST13 and IS*903B-mcr-9.1-wbuc*-IS*26* in ST256 strains are probably to be associated with IncFIB and IncHI2 plasmids, respectively (Fig. 1). IncFIB type plasmid has been predicted as the virulence plasmid of *C. sakazakii* (1, 6, 36). IncHI2 plasmids have been identified as important carriers of drug resistance genes in other pathogenic bacteria, and their close association with *mcr-9.1* is also confirmed (37–39). In this study, the *mcr-9.1*-positive IncHI2 plasmid pGW1 was also a drug resistance plasmid, and its flanking structure belonged to the type 3 *mcr-9* backbone which was prevalent in many genera of bacteria (1, 12). More importantly, the *mcr-9.1*-positive IncHI2 plasmid pGW1 in *C. sakazakii* GZcsf-1 can be transferred to *E. coli* J53, conferring multiple drug resistance (Table 2). The *mcr-9.1*-positive conjugative plasmid in *C. sakazakii* strains suggests a high risk for its rapid dissemination in the future.

The *mcr-9.1*-positive strains belonged to specific CTs (Table S1 and Fig. 2). Among ST13 strains, the *mcr-9.1*-positive CT212 clinical strain bq exhibited closest phylogenetic relatedness to CT53 strain C.18 (source: milk powder) (Table S2). As for ST256 strains, *mcr-9.1*-positive CT222 clinical strains GZcsf-1 and CRZK had the closest relationship with CT223 environmental strain. While ST256 has not been detected in any contaminated food, its presence in insect and environmental samples suggests a potential transmission route requiring further investigation. The powdered infant formula (PIF) is thought to be the major transmission vehicle, but its transmission dynamics are poorly understood. A recent study reported that approximately 1/4 of households in the United States are contaminated with *C. sakazakii*, with kitchens being a particularly prevalent setting for this contamination (40). It may contribute to contamination of PIF and provides insights into mitigating the risk of transmission (40).

In this study, *C. sakazakii* ST13 strains in lineages I and II exclusively harbored type 1 ancestral spacers. Within lineage III, strains 1915004 and 1915005—positioned at phylogenetically peripheral regions—retained both type 1 and type 2 ancestral spacers, whereas all other lineage III strains solely carried type 2 ancestral spacers (Fig. 4). Crucially, spacers sak1-79 (from type 2 ancestral spacers) and sak1-750 (at the type 1 and type 2 ancestral spacer junction, unique in strains 1915004 and 1915005) exhibited sequence homology to distinct phage genomes (Fig. 3), strongly implicating phage predation as a key driver of the evolutionary divergence. Evolutionary trajectory analysis indicated ancestral *C. sakazakii* ST13 strains initially carried type 1 ancestral spacers (Fig. 4). Prolonged phage pressure drove adaptive diversification, leading to lineage III with the coexistence of both type 1 and type 2 ancestral spacers. Subsequent evolutionary selection favored type 2 retention (e.g., dominant foodborne strain CT208), while type 1 counterparts were eliminated. Notably, while *mcr-9.1*-positive strains were distributed across all three lineages, lineage III contained both clinical *mcr-9.1*-positive CT212 and major foodborne CT208 isolates, suggesting its role in a high-risk cluster warranting prioritized surveillance. Despite the excellent discriminatory power of CRISPR typing in distinguishing similar *Cronobacter* strains, its widespread application is limited due to the lack of integrated typing tools and publicly accessible databases. In the future, our team will be dedicated to developing such tools to enable researchers globally to easily utilize this typing method.

In conclusion, this study systematically investigates the prevalence, flanking genetic structures, and horizontal transfer capacity of *mcr-9.1* gene in clinical *C. sakazakii* strains isolated from two Chinese hospitals. Furthermore, we delineated the evolutionary trajectories of *mcr-9.1*-positive ST13 and ST256 strains through integrated analysis of CRISPR diversity and wgSNP. Our findings demonstrate that CRISPR typing outperforms traditional methods in resolving fine-scale epidemiological relationships, thereby proposing its prioritization for source tracking in clinical infections and foodborne contamination investigations. The distribution of *mcr-9.1* in conjugative plasmids of pathogenic bacteria may be a threat to public health, thereby warranting clinical monitoring.

## Data Availability

The GenBank accession numbers of *C. sakazakii* bq (SAMN44049741) are CP170808 and CP170809, and those of *C. sakazakii* CRZK (SAMN44049244) and BD (SAMN44049245) are JBKFVP000000000 and JBKFVO000000000.

## SUPPLEMENTAL MATERIAL

Table S1 aem.01379-25-s0001.docx Antimicrobial drug susceptibility profiles of clinical *Cronobacter* species strains collected in this study.

Table S2 aem.01379-25-s0002.docx Information of all *C. sakazakii* ST13 and ST256 strains used in this study.

Table S3 aem.01379-25-s0003.docx Code of CRISPR types (CTs), order of CRISPR array spacers, and dictionary of CRISPR spacers in *C. sakazakii* ST13 and ST256 strains from this study.

Table S4 aem.01379-25-s0004.docx CRISPR spacers in *C. sakazakii* ST13 and ST256 strains matching phage and plasmid sequences.

ASM does not own the copyrights to Supplemental Material that may be linked to, or accessed through, an article. The authors have granted ASM a non-exclusive, world-wide license to publish the Supplemental Material files. Please contact the corresponding author directly for reuse.
