# Supplementary material for: Emergence of transmissible mcr-9.1 plasmids in clinical Cronobacter sakazakii: CRISPR typing unravels phage-driven evolution and high-risk lineage
Source: Appl Environ Microbiol. 2025 Sep 2;91(10):e01379-25. doi: 10.1128/aem.01379-25 (PMC12542685; doi:10.1128/aem.01379-25)
Supplement: Table S1 — Antimicrobial drug susceptibility profiles of clinical Cronobacter species strains collected in this study. [file aem.01379-25-s0001.docx]

Table S1. Antimicrobial drug susceptibility profiles of clinical *Cronobacter* spp. strains collected in this study.

| Antimicrobial group | Antibiotic | Antimicrobial susceptibility^#^ | | | | |  |  |
| --- | --- | --- | --- | --- | --- | --- | --- | --- |
|  |  | GZcsf-1 | CRZK | BD | GZfs | bq | H5 | H6 |
| Penicillins | Ampicillin | R | R | R | S | S | I | I |
|  | Ampicillin/sulbactam | R | R | S | S | S | S | S |
|  | Amoxicillin/Clavulanic | R | R | S | S | S | R | R |
|  | Piperacillin/Tazobactam | S | S | S | S | S | S | S |
| Cephalosporins | Cefepime | S | S | S | S | S | S | S |
|  | Ceftriaxone | R | R | R | S | S | S | S |
|  | Cefazolin | R | R | R | S | S | R | R |
|  | Ceftazidime | S | S | S | S | S | S | S |
|  | Cefuroxime | R | R | R | R | S | S | S |
| Aminoglycosides | Tobramycin | I | I | S | S | S | S | S |
|  | Gentamicin | R | R | S | S | S | S | I |
| Quinolones | Ciprofloxacin | S | S | S | S | S | S | S |
| Carbapenems | Imipenem | S | S | S | S | S | S | S |
| Sulfonamides | Trimethoprim/sulfamethoxazole | R | R | S | S | S | S | S |
| Monobactams | Aztreonam | R | R | R | S | S | S | S |
| Amphenicols | Chloramphenicol | R | R | S | S | S | S | S |
| Tetracyclines | Tigecycline | S | S | S | S | S | S | S |
|  | Tetracycline | R | R | S | S | S | S | S |
| Lipopepetide | Colistin | S | S | S | S | S | S | S |

^#^ The colistin and tigecycline resistance were defined using the *Enterobacteriaceae* data from the Clinical and Laboratory Standards Institute (CLSI) breakpoints of minimum inhibitory concentrations (MIC) interpretive criteria, other results are interpreted using zone diameter (KB) interpretive criteria.
