# Supplementary material for: Emergence of transmissible mcr-9.1 plasmids in clinical Cronobacter sakazakii: CRISPR typing unravels phage-driven evolution and high-risk lineage
Source: Appl Environ Microbiol. 2025 Sep 2;91(10):e01379-25. doi: 10.1128/aem.01379-25 (PMC12542685; doi:10.1128/aem.01379-25)
Supplement: Table S2 — Information of all C. sakazakii ST13 and ST256 strains used in this study. [file aem.01379-25-s0002.docx]

Table S2. The information of all *C. sakazakii* ST13 and ST256 strains used in this study

| isolate | accession number or id* | public database | year | country | source | ST | CT | *mcr-9.1* |
| --- | --- | --- | --- | --- | --- | --- | --- | --- |
| 693 | 1136 | *Cronobacter* PubMLST database | 1994 | France | Clinical | 13 | 52 | - |
| 700 | 1140 | *Cronobacter* PubMLST database | 1994 | France | Clinical | 13 | 52 | - |
| 713 | 1149 | *Cronobacter* PubMLST database | 1994 | France | Food_Infant formula | 13 | 52 | - |
| 714 | 1151 | *Cronobacter* PubMLST database | 1994 | France | Food_Infant formula | 13 | 52 | - |
| 715 | 1152 | *Cronobacter* PubMLST database | 1994 | France | Food_Infant formula | 13 | 52 | - |
| C.18 | 2696 | *Cronobacter* PubMLST database | 2006 | China | Food_Milk powder | 13 | 53 | - |
| CS-1 | 1952 | *Cronobacter* PubMLST database | 2005 | USA | Food_Food ingredient | 13 | 208 | - |
| CS-9 | 1958 | *Cronobacter* PubMLST database | 2005 | New Zealand | Food_Food ingredient | 13 | 208 | - |
| CS-14 | 1961 | *Cronobacter* PubMLST database | 2005 | USA | Food_Milk powder | 13 | 208 | - |
| CS-18 | 1964 | *Cronobacter* PubMLST database | 2005 | New Zealand | Food_Milk powder | 13 | 208 | - |
| CS-19 | 1965 | *Cronobacter* PubMLST database | 2005 | New Zealand | Food_Milk powder | 13 | 208 | - |
| CS-25 | 1967 | *Cronobacter* PubMLST database | 2005 | New Zealand | Food_Infant formula | 13 | 208 | - |
| CS-30 | 1968 | *Cronobacter* PubMLST database | 2005 | New Zealand | Food_Infant formula | 13 | 208 | - |
| CS-32 | 1969 | *Cronobacter* PubMLST database | 2005 | New Zealand |  | 13 | 208 | - |
| CS-33 | 1970 | *Cronobacter* PubMLST database | 2006 | France | Food | 13 | 208 | - |
| CS-34 | 1971 | *Cronobacter* PubMLST database | 2006 | Australia | Food | 13 | 208 | - |
| CS-36 | 1972 | *Cronobacter* PubMLST database | 2006 | New Zealand | Food_Infant formula | 13 | 208 | - |
| CS-37 | 1973 | *Cronobacter* PubMLST database | 2006 | Australia | Food | 13 | 208 | - |
| CS-38 | 1974 | *Cronobacter* PubMLST database | 2006 | Australia | Food | 13 | 208 | - |
| CS-45 | 1978 | *Cronobacter* PubMLST database | 2006 | New Zealand | Food_Infant formula | 13 | 208 | - |
| CS-47 | 1979 | *Cronobacter* PubMLST database | 2006 | France | Food_Food ingredient | 13 | 208 | - |
| CS-48 | 1980 | *Cronobacter* PubMLST database | 2006 | New Zealand | Food_Infant formula | 13 | 208 | - |
| CS-52 | 1983 | *Cronobacter* PubMLST database | 2006 | New Zealand | Food_Infant formula | 13 | 208 | - |
| CS-56 | 1987 | *Cronobacter* PubMLST database | 2006 | New Zealand | Food_Milk powder | 13 | 208 | - |
| CS-60 | 1990 | *Cronobacter* PubMLST database | 2006 | New Zealand | Food_Milk powder | 13 | 208 | - |
| CS-61 | 1991 | *Cronobacter* PubMLST database | 2006 | New Zealand | Food_Milk powder | 13 | 208 | - |
| CS-63 | 1993 | *Cronobacter* PubMLST database | 2006 | USA |  | 13 | 208 | - |
| CS-64 | 1994 | *Cronobacter* PubMLST database | 2006 | Belgium | Food_Food ingredient | 13 | 208 | - |
| CS-65 | 1995 | *Cronobacter* PubMLST database | 2006 | USA | Food_Food ingredient | 13 | 208 | - |
| CS-66 | 1996 | *Cronobacter* PubMLST database | 2006 | USA | Food_Food ingredient | 13 | 208 | - |
| CS-72 | 2000 | *Cronobacter* PubMLST database | 2006 | New Zealand | Food_Infant formula | 13 | 208 | - |
| wls2208 | 3468 | *Cronobacter* PubMLST database |  |  |  | 13 | 208 | - |
| CS-8 | 1957 | *Cronobacter* PubMLST database | 2005 | New Zealand | IFood_nfant formula | 13 | 209 | - |
| CS-44 | 1977 | *Cronobacter* PubMLST database | 2006 | France | Food | 13 | 209 | - |
| CS-17 | 1963 | *Cronobacter* PubMLST database | 2005 | Australia | Food_Food ingredient | 13 | 210 | - |
| CS-62 | 1992 | *Cronobacter* PubMLST database | 2006 | USA | Food_Food ingredient | 13 | 210 | - |
| C.20 | 2698 | *Cronobacter* PubMLST database | 2006 | China | Food_Powdered infant formula | 13 | 211 | - |
| C.21 | 2699 | *Cronobacter* PubMLST database | 2006 | China | Food_Powdered infant formula | 13 | 211 | - |
| bq | CP170808/CP170809 | NCBI GenBank | 2022 | China_Guangzhou | Clinical_urine | 13 | 212 | + |
| EUCRONI016 | 2505 | *Cronobacter* PubMLST database | 2016 | Belgium | Clinical | 13 | 213 | - |
| FDA1033055-S055-002 | 3419 | *Cronobacter* PubMLST database | 2022 | USA | Environmental | 13 | 213 | - |
| 1887 | 430 | *Cronobacter* PubMLST database | 2011 | Czech Republic | Food | 13 | 213 | - |
| CFSAN019574 | 1321 | *Cronobacter* PubMLST database | 2014 | USA | Environmental | 13 | 214 | - |
| CS-71 | 1999 | *Cronobacter* PubMLST database | 2006 | France | Food_Milk powder | 13 | 215 | - |
| MOD1_LR757 | 2430 | *Cronobacter* PubMLST database | 2008 | USA | Food_Food ingredient | 13 | 216 | - |
| ZV-2696-17 | 2595 | *Cronobacter* PubMLST database | 2017 | Slovenia | Food_Food ingredient | 13 | 217 | - |
| ZV-3091-16 | 2596 | *Cronobacter* PubMLST database | 2016 | Slovenia | Food_Food ingredient | 13 | 218 | - |
| MOD1_KW13 | 2660 | *Cronobacter* PubMLST database |  | Switzerland | Food_Spice | 13 | 218 | - |
| CQ04 | 2958 | *Cronobacter* PubMLST database | 2012 | China | Food | 13 | 218 | - |
| HA18040 | 3000 | *Cronobacter* PubMLST database | 2020 | China_zhengzhou | Food_Powdered infant formula | 13 | 218 | + |
| 10406 | 10406 | the National Genomics Data Center, Beijing Institute of Genomics | 2006 | China_Liaoning | Food_Grain food products | 13 | 219 | + |
| 10456 | 10456 | the National Genomics Data Center, Beijing Institute of Genomics | 2008 | China_Liaoning | Food_Grain food products | 13 | 219 | + |
| 10457 | 10457 | the National Genomics Data Center, Beijing Institute of Genomics | 2008 | China_Liaoning | Food_Grain food products | 13 | 219 | + |
| 10469 | 10469 | the National Genomics Data Center, Beijing Institute of Genomics | 2008 | China_Liaoning | Food_Milk dairy products | 13 | 219 | + |
| 1915004 | 3226 | *Cronobacter* PubMLST database | 2019 | China_wenzhou | Food_Food ingredient | 13 | 220 | - |
| SCCR0024 | SCCR0024 | the National Genomics Data Center, Beijing Institute of Genomics | 2010 | China_Shandong | Food_Beverages | 13 | 221 | + |
| GZcsf-1 | 2635 | *Cronobacter* PubMLST database | 2015 | China_Guangzhou | Clinical_brain abscess fluid | 256 | 222 | + |
| CRZK | JBKFVP000000000 | NCBI GenBank | 2015 | China_Guangzhou | Clinical_blood | 256 | 222 | + |
| Crono15_YL | 3204 | *Cronobacter* PubMLST database | 2016 | China | Environmental | 256 | 223 | - |
| AM65_5MHA | 3820 | *Cronobacter* PubMLST database | 2014 | China_Shenzhen | Clinical_fecal material | 256 | 224 | - |
| CS-2 | 1953 | *Cronobacter* PubMLST database | 2005 | The Netherlands | Food_Infant formula | 13 |  | - |
| CS-6 | 1956 | *Cronobacter* PubMLST database | 2005 | New Zealand | Food_Infant formula | 13 |  | - |
| 1915005 | 3227 | *Cronobacter* PubMLST database | 2019 | China_wenzhou | Food_Food ingredient | 13 |  | - |
| MOD1_Ls15g | 1923 | *Cronobacter* PubMLST database | 2012 | USA | Insect | 256 |  | - |

* The accession number or id of the isolate in the corresponding database.
