# Supplementary material for: Emergence of transmissible mcr-9.1 plasmids in clinical Cronobacter sakazakii: CRISPR typing unravels phage-driven evolution and high-risk lineage
Source: Appl Environ Microbiol. 2025 Sep 2;91(10):e01379-25. doi: 10.1128/aem.01379-25 (PMC12542685; doi:10.1128/aem.01379-25)
Supplement: Table S3 — Code of CRISPR types (CTs), order of CRISPR array spacers, and dictionary of CRISPR spacers in C. sakazakii ST13 and ST256 strains from this study. [file aem.01379-25-s0003.docx]

Table S3. The code of CRISPR types (CTs), the order of CRISPR array spacers, and the dictionary of CRISPR spacers in *C. sakazakii* ST13 and ST256 strains of this study

① CTs code

| CT | CRISPR1-Serial number | CRISPR2-Serial number | CRISPR3-Serial number | CRISPR6-Serial number |
| --- | --- | --- | --- | --- |
| 52 | 33 | 50 |  |  |
| 53 | 33 | 51 |  |  |
| 208 | 143 | 51 |  |  |
| 209 | 144 | 51 |  |  |
| 210 | 145 | 51 |  |  |
| 211 | 150 | 51 |  |  |
| 212 | 152 | 51 |  |  |
| 213 | 141 | 160 |  |  |
| 214 | 142 | 161 |  |  |
| 215 | 146 | 160 |  |  |
| 216 | 147 | 162 |  |  |
| 217 | 148 | 163 |  |  |
| 218 | 149 | 164 |  |  |
| 219 | 149 | 166 |  |  |
| 220 | 151 | 165 |  |  |
| 221 | 153 | 162 |  |  |
| 222 | 154 | 167 | 67 |  |
| 223 | 155 | 167 | 67 |  |
| 224 | 156 | 168 | 67 |  |

|  |
| --- |

② the order of CRISPR array spacers

| CRISPR1-Serial number | CRISPR1-spacers-order (key-order) |
| --- | --- |
| 33 | 91,90,89,88,88,87,391,86,85,84,83,82,81,80,79 |
| 141 | 91,88,88,87,391,86,392,136,135,134 |
| 142 | 91,90,394,393,87,391,85,392,136,135,134, |
| 143 | 91,90,89,88,88,87,391,86,85,84,83,80,79 |
| 144 | 91,90,89,88,88,88,87,391,86,85,84,83,80,79 |
| 145 | 91,90,89,88,87,391,86,85,84,83,80,79 |
| 146 | 91,90,88,87,391,86,392,136,135,134 |
| 147 | 393,89,88,87,391,86,749,85,84,83,136,135,134 |
| 148 | 91,90,394,393,89,88,88,87,391,86,85,84,83,82,81,80,79 |
| 149 | 91,90,394,393,88,87,391,85,392,136,135,134, |
| 150 | 83,82,81,80,79 |
| 151 | 91,90,394,393,89,88,87,391,86,85,84,83,80,79,751,750,136,135,134 |
| 152 | 91,90,394,393,89,88,88,87,86,85,84,82,81,80,79 |
| 153 | 393,89,88,87,391,86,749,85,84,136,135,134 |
| 154 | 806,805,804,803,802,801,800,799,798,797,796,795,794,793,792,791,790,789,788,787,786,785,784,783,782,781,780,779,778,777,776,775,774,773,772,771,770,769,768,767,766,765,764,763,762,761,760,759,758,757,756,262,261,715,260,733,715,260,733,754,753,258,257,256,253,102 |
| 155 | 806,805,804,803,802,801,800,799,798,793,792,791,790,789,788,787,786,785,784,783,782,781,780,779,778,777,776,775,774,773,772,771,770,769,768,767,766,765,764,763,762,761,760,759,758,757,756,262,261,715,260,733,715,260,733,754,753,258,257,256,253,102 |
| 156 | 806,805,804,803,802,801,800,799,798,797,796,795,794,793,792,791,790,789,788,787,786,785,784,783,782,781,780,779,778,777,776,775,774,773,772,771,770,769,768,767,766,765,764,763,762,761,760,759,758,757,756,262,261,715,260,733,715,260,733,259,754,753,258,257,256,253,102 |

| CRISPR2-Serial number | CRISPR2-spacers-order (key-order) |
| --- | --- |
| 50 | 114,113,112,111,110,109,108,101 |
| 51 | 114,113,112,111,110,109,108,431,430,101 |
| 160 | 114,459,537,113,112,111,110,109,108,458,431,430,101 |
| 161 | 114,459,537,112,431,430,101,101 |
| 162 | 114,537,113,112,111,110,109,108,458,457,456,431,430,101 |
| 163 | 114,113,111,110,109,108,458,457,456,431,430,101 |
| 164 | 114,459,537,113,112,431,430,101 |
| 165 | 114,113,112,111,110,109,108,458,457,456,431,430,101 |
| 166 | 114,459,113,112,431,430,101 |
| 167 | 896,895,894,893,892,319,891,890,889,888,887,886,885,884,883,822 |
| 168 | 896,895,894,893,892,319,891,890,889,888,887,886,885,884,883,901,900,899,898,897,823,822 |

| CRISPR3-Serial number | CRISPR3-spacers-order (key-order) |
| --- | --- |
| 67 | 365,364,342 |

③ the dictionary of CRISPR spacers

Dictionary of CRISPR1 spacers

| key | AnnotatedSpacer | sequence |
| --- | --- | --- |
| 79 | sak1-79 | ATTTCAGTGTCTTCGTCCGTCAGCGCACCTTC |
| 80 | sak1-80 | TCGATCGGCAGGTGGCCGAGCGCAGCGAGAAA |
| 81 | sak1-81 | GTCACTCAGGGATTCCGAAAGCCACGAAAGCG |
| 82 | sak1-82 | GTGAGCTTGTTGGCGCGGCATGGTATGAGCTG |
| 83 | sak1-83 | GCTGCGCTTGGCCTGCAATATATCGGTACACA |
| 84 | sak1-84 | CCGAACATGACCTGATGCGCCTGGCTATCGAT |
| 85 | sak1-85 | AGCAAAATAGATAACGCACAGAACGAAAACCT |
| 86 | sak1-86 | GGGGCGGTGTTGTTCTTTAGCGCGAAGCGCTG |
| 87 | sak1-87 | CGTCCAGCAGAGACAGCAGAAACGGCAGCAGT |
| 88 | sak1-88 | ATGGATTTTATCGACCGCCCAATTGATTATTA |
| 89 | sak1-89 | AGCTCAGCGGCGCAATTATCGATTTCCGCGGC |
| 90 | sak1-90 | TGCTCTCGTTAGATTTAAACCAGCTGACCAGG |
| 91 | sak1-91 | CATTTTCTTTATCACACAACTTTAATTGCCCT |
| 102 | sak1-102 | GTTCTTTAACGCTCTGGTGACGCTTACCGTAT |
| 134 | sak1-134 | ATTCACGGTAACGTCACCGCCAGCACCGTTTTG |
| 135 | sak1-135 | CGACACGATCCGCCGCCTGGGTTACGAAAGC |
| 136 | sak1-136 | CCAATCATTAAAACATAAGGAACCACCTATGA |
| 253 | sak1-253 | ATTGCGGGATGACCAGTTCGCGAGCTTTCTGA |
| 256 | sak1-256 | CCGCCATCATGCGGCTCACTTGATGCGGATGA |
| 257 | sak1-257 | AGACCAGACGCCGATACCAGCGAAGAAATGGC |
| 258 | sak1-258 | AACGTGTAAATCAACTGGAGGCACGGGTCAAA |
| 259 | sak1-259 | GCGAATCAAAAGGAAAAAAATATAACGAAATC |
| 260 | sak1-260 | GGTCGACACGCCCGCCGAACGTCACGGTTGAT |
| 261 | sak1-261 | TATACGCCCCGAGTCCCGTTTTTGCGCCGTAG |
| 262 | sak1-262 | ACGTCTTCGACCCATTTATCCGTCGCGGCGCT |
| 391 | sak1-391 | ACAAGCCGCAGCGGCGGCGGGGGGTTATTTTTG |
| 392 | sak1-392 | CCGGCAGCGCCGTAAAAAGGCGCGCTGATTGC |
| 393 | sak1-393 | TTACCCCCGCATCCATTGAACCGCTTTTAAAA |
| 394 | sak1-394 | CTGACACTGACGATAGTGAGGAGGTAGACGAG |
| 715 | sak1-715 | GCTGCTATCTCGGGTATTTACGACGCGATCCA |
| 733 | sak1-733 | TCGTTTTTGCGGTTATTGTTGCTCACCTGTTG |
| 749 | sak1-749 | CTGTCAATGACACTGGCTCCCCGACCTTAACG |
| 750 | sak1-750 | AATACGCCTGGATTGCTGACTTCACTGACTCG |
| 751 | sak1-751 | CCGTCGGCATTGGTATCTAAACCGCGATTTGT |
| 753 | sak1-753 | ATTAACGGACTGCAAACTAAACCGTTTTTAAC |
| 754 | sak1-754 | CGCGCCGGCGCTACCAGTTGTATTGCTTGCTG |
| 756 | sak1-756 | CGGGTGTTTATCCGCGTTCAGTCGGAATTTGA |
| 757 | sak1-757 | TGCCGATGGCGGTCTACTGCGCGCCGCTCGTT |
| 758 | sak1-758 | CAGATTCCGTGACTTACATAATCCCCAGCCAC |
| 759 | sak1-759 | GTTATCGGATTCAACCCTTCATCAGCAAGGTG |
| 760 | sak1-760 | TTTTTAAGAGCGGGGCTAACCACAACGGCGTT |
| 761 | sak1-761 | CCTGCCAAACCATGCAGGCTTCACCCTCCATG |
| 762 | sak1-762 | TTTTTAACTCATTTACAAGGTGGCTCTCAACC |
| 763 | sak1-763 | TCAGACGCGGGTACGGTCGTAGTGTTCTGCTC |
| 764 | sak1-764 | GCCGATGAGGCCAACAAACAGGAGTGATCATG |
| 765 | sak1-765 | GATGCGCGCGCGCTGGGACGAACGCAGCGCCC |
| 766 | sak1-766 | ACCAGCCACCGTGTCACTACTGTGGAAGGTAT |
| 767 | sak1-767 | GCTATATCCACGCTGCGGCCAATAGCCATTTC |
| 768 | sak1-768 | GGGTGAGGACGTTCAACCGAAGCATATAGGAG |
| 769 | sak1-769 | GTCGTCAGGGCTGTGCAGTCAGCGCTGCTAAT |
| 770 | sak1-770 | CACCCGAATGCGTTGTCTTTTGAGTTGAGGAC |
| 771 | sak1-771 | ATCCGGCTCACTGGCAAATCGTGGACACAGAC |
| 772 | sak1-772 | CGTTCAGCCGGAATAATTTTTGTTTCAGTGGT |
| 773 | sak1-773 | TGCTGTTACCCCATACATTAGGGTTTGACGCC |
| 774 | sak1-774 | GTTTTACCAACGCTGCCACTATAATTAATTAC |
| 775 | sak1-775 | CTCCTCAGATTAGGCCCGACGCCTTGCGGCGG |
| 776 | sak1-776 | GCGATCATCAGGTAGAAATTCGTTGCAGAGAA |
| 777 | sak1-777 | CGTCTGACGCCGGGGCGTATCATCATGACCGA |
| 778 | sak1-778 | ATAATCGAATCCCCATGATGATGTTAGGTGGT |
| 779 | sak1-779 | ACGGCGGCGTTGTTGTGATTGCAGCGTTTGAT |
| 780 | sak1-780 | TTTGCAATGATGTTTTTCAGGGCCTCTTTCTT |
| 781 | sak1-781 | GGCGGAGAATTTACATTTTTCAATAGCTGGTA |
| 782 | sak1-782 | TGGTCATTACGCTCTCCTTGTGGTGTCTGAGA |
| 783 | sak1-783 | TATTCTCATTGATCTCTTTTGAGTTCATCTTT |
| 784 | sak1-784 | TCGGCCTCGTTCTGCTGATAGAACGCCTCGGC |
| 785 | sak1-785 | TGTCGACCCTGCAGTTCTTTACGATGCTTCGT |
| 786 | sak1-786 | ATTCAACGCGCGTGCGCAGCTCAACAGCCGTC |
| 787 | sak1-787 | ATTCAACACAATCCGCCCCATCCGGGGCAAAT |
| 788 | sak1-788 | CGGCCACTCAGAACGAGTTCAACGCCGTCATA |
| 789 | sak1-789 | ACGTGGTAATTACCCCGGCATTTAGCGGTGAC |
| 790 | sak1-790 | AACCCGCCGGAAGTGGGAAAACTTTCGCTGCC |
| 791 | sak1-791 | GCCTCCCATACCGGAATAAGCCGCACGATGGC |
| 792 | sak1-792 | CTCCGCGTAATCCATCAACTGCTCGTGCCACT |
| 793 | sak1-793 | GCCGGTTTTTGCTTTTGTTGCCACTGTTGAAAC |
| 794 | sak1-794 | CGTATATGGACATGAATTAGGTGCTGATTATG |
| 795 | sak1-795 | TCAGACGCGGGTACGGTCGTAGTGTTCTGCTC |
| 796 | sak1-796 | TTTTTAACTCATTTACAAGGTGGCTCTCAACC |
| 797 | sak1-797 | CCTGCCAAACCATGCAGGCTTCACCCTCCATG |
| 798 | sak1-798 | TTTTTAAGAGCGGGGCTAACCACAACGGCGTT |
| 799 | sak1-799 | GTTATCGGATTCAACCCTTCATCAGCAAGGTG |
| 800 | sak1-800 | CAGATTCCGTGACTTACATAATCCCCAGCCAC |
| 801 | sak1-801 | TGCCGATGGCGGTCTACTGCGCGCCGCTCGTT |
| 802 | sak1-802 | CGGGTGTTTATCCGCGTTCAGTCGGAATTTGA |
| 803 | sak1-803 | TCGTTTTTGCGGTTATTGTTGCTCACCTGTTG |
| 804 | sak1-804 | CGCGCCGGCGCTACCAGTTGTATTGCTTGCTG |
| 805 | sak1-805 | ATTAACGGACTGCAAACTAAACCGTTTTTAAC |
| 806 | sak1-806 | CCGCCATCATGCGGCTCACTTGATGCGGATGA |

Dictionary of CRISPR2 spacers

| key | AnnotatedSpacer | sequence |
| --- | --- | --- |
| 101 | sak2-101 | TACGGCGCTTGCAGCTCTTGCGATCATTATGG |
| 108 | sak2-108 | TCGGAATCCTATGGTCAGCCTGTGATGTACAA |
| 109 | sak2-109 | ACCCGCCAGGCTGATGAACTCGCAGACCGGCA |
| 110 | sak2-110 | GTGTTAACTCAAATTCATCGTCCCACGGTGGG |
| 111 | sak2-111 | AAGGCGTTTACGGCTTGTTGGGCTTGTTTAAT |
| 112 | sak2-112 | CCTCCTCATGCGAGCGGGCGCTGGTCATCAGC |
| 113 | sak2-113 | GCTGCAATGCCGGTCAGGGTCAGCAGTTCCTG |
| 114 | sak2-114 | CTGGTCAAAGCCGCACAAAAATCCCATGTCGA |
| 430 | sak2-430 | TCTTCCAGGAGATCGATCCCTTGTCTCAGAGC |
| 431 | sak2-431 | TTTGCCACCACAATAATCATTCCAGTTCTCAT |
| 456 | sak2-456 | CTCACACTGATTCCATTCAACGCAACATAGGA |
| 457 | sak2-457 | CGGAATACAAAATTTTCGTCAGCGTTCGCTGT |
| 458 | sak2-458 | CAGTCGGAGAAAGGTGCGCGGTTTATTGAATT |
| 459 | sak2-459 | CGCAACGCGCTGCACCTGACCAGACAATTTTT |
| 537 | sak2-537 | CCTTCGCTATCGATCACGCTCAGATGGGAATC |
| 822 | sak2-822 | GCGGTGCCGCATGACCGACACAGCACAACTGA |
| 883 | sak2-883 | GGTGCATCCGGCGGCAGCGGCTGGCGCATTCA |
| 884 | sak2-884 | TTAGTTGACACGTATTTACCGGGCGTTATCGA |
| 885 | sak2-885 | GGAAGCGCCGTGCGCTGTGCGATGGGAAGCGC |
| 886 | sak2-886 | GCTGGGAAAGTGGGGTTGTCACAGCGTGTCAC |
| 887 | sak2-887 | CGAGGGGGCTGTCAAGGGGATTTATTGTGCTG |
| 888 | sak2-888 | TTTCCGCCGGTCGCGGCGTCGTATACCGCAAA |
| 889 | sak2-889 | TCACCTCTCTCTTTTTTTTGAATAAAATCACC |
| 890 | sak2-890 | GCAACGAGAACAAAATTGATGGCGCGGTCGCG |
| 891 | sak2-891 | GCGGTTGCACTCCACCACGAACCCCCCGAACC |
| 892 | sak2-892 | TCCACTTGAGAGCAGAGCCCGACGCCAGAAAT |
| 893 | sak2-893 | GTGGTGAAAACCTTTTTAATTGCCGTAGCGGC |
| 894 | sak2-894 | CGAGGCTTTGCGTCACTAAAAAAATTGGGGCT |
| 895 | sak2-895 | CACGACCGGCCGGGATCGTAACGGTTAAAGGC |
| 896 | sak2-896 | CACACAAAAACACGCAGCAACAATATTTTCAA |
| 897 | sak2-897 | GTAATACTATGAGGGTTACTTGCCCGGCGCGC |
| 898 | sak2-898 | GCAGCGGCAGAACAAGGCGAATCCGCGGGGGT |
| 899 | sak2-899 | CGTATAGATTTCCCCTATAACTGGCGCATTTG |
| 900 | sak2-900 | CGGCCACAGGGAGCGGCGCGGCCATACCTCAG |
| 901 | sak2-901 | GGGAGGTTCTTACTGATATTTCAAATATTAAC |

Dictionary of CRISPR3 spacers

| key | AnnotatedSpacer | sequence |
| --- | --- | --- |
| 342 | sak2-342 | TCCGGCCTCCGGCCTCCGCGCTCCGCTTGGCC |
| 364 | sak2-364 | TGCTGGCGCATCCGTTCGCTAAACTGCCTGTC |
| 365 | sak2-365 | TATTGCCGGTGGAACAATGACCGGGATGATAG |
