# Supplementary material for: Emergence of transmissible mcr-9.1 plasmids in clinical Cronobacter sakazakii: CRISPR typing unravels phage-driven evolution and high-risk lineage
Source: Appl Environ Microbiol. 2025 Sep 2;91(10):e01379-25. doi: 10.1128/aem.01379-25 (PMC12542685; doi:10.1128/aem.01379-25)
Supplement: Table S4 — CRISPR spacers in C. sakazakii ST13 and ST256 strains matching phage and plasmid sequences. [file aem.01379-25-s0004.docx]

Table S4. CRISPR spacers in *C. sakazakii* ST13 and ST256 strains matching phage and plasmid sequences.

| CRISPR array | AnnotatedSpacer | Target | Region | Annotation | Accession number |
| --- | --- | --- | --- | --- | --- |
| ST13 CRISPR1 | sak1-79 | *Enterobacter* phage BUCT554 | 6558-6589 | terminase large subunit | MW205203.1 |
|  | sak1-750 | *Enterobacter* phage vB_EclS_CobraSix | 5159-5190 | hypothetical protein | NC_071003.1 |
| ST256 CRISPR1 | sak1-762 | *E. coli* strain 7/2 plasmid p7_2.2 | 35947-35978 | hypothetical protein | CP023822.1 |
|  | sak1-766 | *C. sakazakii* strain MOD1-GK1025B plasmid pGK1025B_3 | 6197-6228 | conjugative relaxase | CP078109 |
|  | sak1-768 | *E. coli* strain 7/2 plasmid p7_2.2 | 31093-31122 | hypothetical protein | CP023822.1 |
|  | sak1-774 | *C. sakazakii* strain MOD1-GK1025B plasmid pGK1025B_3 | 1950-1981 | plasmid stabilization protein | CP078109 |
|  | sak1-776 | *C. sakazakii* strain MOD1-GK1025B plasmid pGK1025B_3 | 35403-35434 | DUF905 domain-containing protein | CP078109 |
|  | sak1-777 | *C. sakazakii* strain MOD1-GK1025B plasmid pGK1025B_3 | 10118-10149 | P-type DNA transfer ATPase VirB11 | CP078109 |
|  | sak1-778 | *C. sakazakii* strain MOD1-GK1025B plasmid pGK1025B_3 | 22647-22678 | hypothetical protein | CP078109 |
| ST256 CRISPR2 | sak2-899 | *Enterobacter roggenkampii* strain WCHER090065 plasmid pMCR10_090065 | 3743-3774 | type IV secretion system DNA-binding domain-containing protein | NZ_CP045065.1 |
